# Dehydroascorbate reductase and monodehydroascorbate reductase activities of two metallothionein-like proteins from sweet potato (*Ipomoea batatas* [L.] Lam. ‘Tainong 57’) storage roots

**DOI:** 10.1186/1999-3110-54-7

**Published:** 2013-08-21

**Authors:** Guan-Jhong Huang, Jeng-Shyan Deng, Hsien-Jung Chen, Shyh-Shyun Huang, Chun-Ching Shih, Yaw-Huei Lin

**Affiliations:** 1 School of Chinese Pharmaceutical Sciences and Chinese Medicine Resources, College of Pharmacy, China Medical University, 404 Taichung, Taiwan; 2 Department of Health and Nutrition Biotechnology, Asia University, 413 Taichung, Taiwan; 3 Department of Biological Sciences, National Sun Yat-sen University, kaohsiung 804, Taiwan; 4 School of Pharmacy, China Medical University, 404 Taichung, Taiwan; 5 Graduate Institute of Pharmaceutical Science and Technology, College of Health Science, Central Taiwan University of Science and Technology, 406 Taichung, Taiwan; 6 Institute of Plant and Microbial Biology, Academia Sinica, 115 Nankang, Taipei, Taiwan

**Keywords:** Sweet potato, Metallothionein, Gene expression, Dehydroascorbate reductase activity, Monodehydroascorbate reductase activity

## Abstract

**Background:**

Metallothionein (MT) is a group of proteins with low molecular masses and high cysteine contents, and it is classified into different types, which generally contains two domains with typical amino acid sequences.

**Results:**

In this report, two full-length cDNAs (MT-1 and MT-II) encoding MT-like proteins were isolated from the roots of sweet potato (*Ipomoea batatas* [L.] Lam. ‘Tainong 57’). Their open reading frames contained 642 and 519 nucleotides (66 and 81 amino acids) for MT-1 and MT-II, respectively, and exhibited a relatively low amino acid sequence similarity. On the basis of the amino acid sequence similarity and conserved residues, it is suggested that MT-I is a member of the plant MT Type-I family, and MT-II is a member of the plant MT Type-II family. The corresponding mRNA levels of MT-1 and MT-II were the highest found in the storage roots. Recombinant MT-1 and MT-II protein overproduced in *E. coli* (M15) was purified by Ni^2+^-chelated affinity chromatography. MT-1 and MT-II reduced dehydroascorbate (DHA) in the presence of glutathione (GSH) to regenerate L-ascorbic acid (AsA). However, without GSH, MT-1 and MT-II has very low DHA reductase activity. And AsA was oxidized by AsA oxidase to generate monodehydroascorbate (MDA) free radical. MDA was also reduced by MT-1 and MT-II to AsA in the presence of NADH mimicking the MDA reductase catalyzed reaction.

**Conclusions:**

These data suggest that MT-1 and MT-II have both DHA reductase and MDA reductase activities. MT-1 and MT-II are apparently the first reported plant MTs exhibiting both DHA and MDA activities *in vitro.*

**Electronic supplementary material:**

The online version of this article (doi:10.1186/1999-3110-54-7) contains supplementary material, which is available to authorized users.

## Background

A variety of plant cell rescue systems adapt to natural environmental conditions by neutralizing toxic reactive oxygen species (ROS). Ascorbate (AsA) plays an important role in various aspects of plants life cycle. AsA regulates growth development such as cell division, cell expansion, and serves as a signal transduction molecule (Pignocchi and Foyer, [2003]). In addition, AsA regeneration system plays an important role in cellular responses and in the defense system against ROS. When AsA functions as an antioxidant in cells, AsA is oxidized into a monodehydroascorbate (MDA) radical in response to the production of excess ROS, after which it is reduced to AsA by MDA reductase (MDAR) (Gill and Tuteja, [2010]). MDA was a sensitive endogenous index of oxidative stress. MDA can in turn non-enzymatically generate AsA and dehydroascorbate (DHA). DHA must be converted to AsA by DHA reductase (DHAR) in the presence of glutathione (GSH) as a reducing agent (Huang et al., [2008]a). Thus, DHAR is a key factor in maintaining a reduced AsA level in the adaptation to environmental conditions.

Metallothioneins (MTs) are proteins of low molecular weights and high cysteine contents with the ability to coordinate heavy metal atoms. Although widely distributed among the animal and plant kingdoms, MTs show extremely heterogeneous compositions (Freisinger, [2011]). Plant MTs generally contain two smaller cysteine-rich domains (4-8 cysteines each) and a large spacer region (30–50 residues) devoid of this amino acid. The distribution of cysteine residues, as well as the length of the spacer region served to further classify plant MTs into four types (Hassinen, et al., [2011]). In plants, most current knowledge on the putative functions of MTs deals with the evaluation of their expression levels, and many physiological roles have been proposed such as metal homeostasis, heavy metal detoxification, oxidative stress response, protection against salinity, carbonate stress, and developmental regulation (Cobbett and Goldsbrough, [2002]). This paper describes cloning, characterization, and biological activities of MT-like proteins (MT-I and MT-II) from sweet potato storage roots. In this study, we also present the evidence to show that the recombination protein, MT-I and MT-II exhibit both DHA reductase and MDA reductase activities.

## Methods

### Materials

Fresh storage roots of sweet potato (*Ipomoea batatas* [L.] Lam. ‘Tainong 57’) were purchased from a local market. After cleaning with water, the roots were placed in a thermostated (28°C) growth chamber and sprayed with water twice a day. Sprouted plants were cultivated in the greenhouse to collect roots, stems, full expanded green leaves, and flowers for experiments. Dehydroascorbate, dehydroascorbate reductase, monodehydroascorbate reductase, ascorbate oxidase, anti-actin (plant) antibody, and other chemicals were purchased from Sigma-Aldrich Chemical Co. (St. Louis, MO, USA).

### PCR-based subtractive hybridization and RACE PCR

Total RNA were isolated separately from the storage roots and sprouts of sweet potato according to the method of Sambrook et al. ([1989]). Then, mRNA was purified with a purification kit (Promega) and used for the differentially-expressed first strand cDNA synthesis using a PCR-based subtractive hybridization kit (Clontech) following the protocol supplied by the manufacturer. The double-strand cDNAs of the storage roots were subtracted by the sprouts, and then ligated to the pGEM-T easy vector for *E. coli* DH5α competent cell transformation. Recombinant plasmids were isolated for DNA sequencing using the ABI PRIZM 337 DNA Sequencer. Nucleotide sequence data were analyzed using the Genetics Computer Group (GCG) programs. Full-length cDNA clone was obtained by performing 5′ and 3′ RACE (5′ and 3′ rapid amplification of cDNA ends) using the Marathon cDNA amplification kit (Clontech) according to the manufacturer’s instructions. The gene-specific primers (MT-1, 5′- TAG GGC CAA AAT AGT GCA AAT T -3′; MT-1I, 5′- GAG ATG CGA AAC TCA GTT GCA A -3′) were used to amplify the double strand cDNAs.

### Expression of MT-I and MT-II proteins in *E. coli*

MT-I and MT-II with its pro-sequence were expressed in *E. coli*. The coding sequence was amplified from cDNA MT-I and MT-II using an oligonucleotide (MT-I, 5′-GGA TCC AGA GAT GTC TTC CGG TTG C -3′; MT-II, 5′-GGA TCC AAA AAT GTC TTG CTG TG-3′), with a *Bam* HI site (underlined) at the putative initial Met residue, and an oligonucleotide (MT-I, 5′- GAC CCT TGC AAC TGT AAG CTT CAA -3′; MT-II, 5′- GCA ATT GCA AGT GAG ATG CGAA G CTT -3′), with a *Hind III* site at the 3′ end. The PCR fragment was subcloned in pGEM T-easy vector. The plasmid was then digested with *Bam* HI and *Hind III* and the excised fragments were subcloned in pQE31 expression vector (QIAexpress expression system, Qiagen). The resulting plasmid, termed pQE-MT-1 and pQE-MT-II respectively, was introduced into *E. coli* (M15). Cultures of the transformed *E. coli* (M15) overexpressed a protein of the expected molecular mass, which was purified by affinity chromatography in Ni-nitrilotriacetic acid (NTA) columns (Qiagen), according to the manufacturer′s instructions.

### RNA isolation and northern blot analysis

Total RNA were extracted from different tissues of sweet potato with TRIzol reagents kit (Invitrogen) according to the manufacturer’s instructions. For northern blotting, 10  μg of total RNA isolated from storage roots, sprouts, sprouted roots, veins, fully expanded green leaves, and flowers were applied to a formaldehyde denaturing gel, then transferred to an Amersham Hybond-N^+^nylon membrane after electrophoresis, according to Sambrook et al. ([1989]). The filter was hybridized sequentially with α-^32^P-labelled defensin full-length cDNA. The procedures for hybridization and autoradiography were according to the Sambrook et al. ([1989]). Visualization of hybridization bands was carried out using X-ray film (Kodak).

### Production of polyclonal antibody and western blot hybridization

Expressed MT-like Y459 (accession no. AF177760) mature protein was cut from the 15% polyacrylamide gel, eluted, and mixed with appropriate amount of pH 7.5 phosphate buffer saline (PBS) containing 0.1% SDS (Chen, et al., [2003]). The eluted proteins were precipitated with acetone containing 10% trichloroacetic acid (TCA) at –20°C for 2 hr. After centrifugation at 13,000 g for 20 min, the pellet was washed with acetone twice, then, dried at room temperature. The acetone powder was re-dissolved in a small amount of PBS containing 0.1% SDS and used as antigens for subcutaneous injections of rabbit to prepare the first antibodies (Taiwan Bio-Pharm Inc.). The second antibody (goat against rabbit Fc portion of Ig) was a product of Sigma-Aldrich (USA). Polyclonal antibodies obtained from rabbit antiserum were utilized for western blot hybridization to study the gene expression of MT-I and MT-II, respectively.

### Electroblotting analysis of MT-I and MT-II proteins

All steps were carried out at 4-8°C. The protein concentration of the supernatant was determined by the Bradford dye-binding assay (Bio-Rad, Hercules, CA). The expression proteins were saved for electroblotting. The crude extract was subjected to 15% SDS–PAGE according to Laemmli ([1970]). After electrophoresis, gels were equilibrated in transfer buffer (25 mM Tris-HCl, pH 8.3, 150 mM glycine and 10% (*w/v*) methanol). The separated proteins were transferred to an Immobilon PVDF membrane (Millipore, Bedford, MA) in transfer buffer at pH 8.3 for 1 hr at 100 V. Membranes were blocked for 2 hr at room temperature in 5% nonfat dry milk powder and then incubated with polyclonal antibody as the primary antibody against MT-I and MT-II proteins. The primary antibody was obtained from rabbit antiserum. After incubation, membranes were washed in phosphate-buffer saline with 0.05% Tween (PBST) three times, 10 min each, then incubated with anti-mouse alkaline phosphatase-conjugated antibody, washed in PBST three times, 10 min each, and developed using NBT (nitro blue tetrazolium)/BCIP (5-bromo-4-chloro- 3-indolyl -phosphate) (Sigma, USA). The secondary antibody (goat against mouse Fc portion of Ig) was a product of Sigma (USA).

### DHA reductase activity assay

The DHA reductase activity of MT-I and MT-II were assayed according to the method of Trümper et al. ([1994]). Ten milligrams DHA were dissolved in 5.0 ml of 100 mM phosphate buffer with pH 6.0 or pH 7.0. The reaction was carried out at 30°C by adding 100 μg MT-I and MT-II solution (100 μg protein) to 0.9 ml DHA solution with or without 4 mM GSH. The increase of absorbance at 265 nm was recorded for 5 min. Non-enzymatic reduction of DHA in phosphate buffer was measured in a separate cuvette at the same time. MT-I or MT-II solution was replaced with empty pQE31-vector proteins for negative controls.

### MDA reductase activity assay

The MDA reductase activity of MT-I and MT-II were assayed according to Hossain et al. ([1984]) by following the decrease in absorbance at 340 nm due to NADH oxidation. MDA free radicals were generated by AsA oxidase (EC 1.10.3.3) in the assay system. The reaction mixtures contained 50 mM phosphate buffer (pH 6.0 or 7.0), 0.33 mM NADH, 3 mM AsA, AsA oxidase (0.9 U), and 200 μL MT-I and MT-II solution (200 μg protein) in a final volume of 1 mL. MT-I or MT-II solution was replaced with empty pQE31-vector proteins for negative controls.

### MDA reductase activity staining in 15% SDS–PAGE gels

MT-I and MT-II were examined for MDA reductase by activity staining in 15% SDS–PAGE gels. Diaphorase activity staining for MDA reductase activity of MT-I or MT-II was according to the methods of Kaplan and Beutler ([1967]) in a 15% SDS–PAGE gel. After electrophoresis, the gel was washed with 25% isopropanol in 10 mM Tris buffer (pH 7.9) twice to remove SDS before activity staining.

### Statistical analysis

Means of triplicate were calculated. Student’s *t* test was used for comparison between two treatments. A difference was considered to be statistically significant when *p* < 0.05.

## Results and discussion

### Isolation and nucleotide sequence of MT-I and MT-II cDNA clones from sweet potato storage roots

MT-I and MT-II cDNA clones from sweet potato storage roots were isolated. We have completed the sequencing of the clones, which were named MT-I and MT-II (MT-1, GenBank Accession Number AF116845 and MT-II, GenBank Accession Number FJ418632). The open reading frames in these two cDNAs encode pro-proteins of 66 and 81 amino acids, respectively, with a predicted molecular mass of 6,614 Da (pI 4.64) and 8,068 Da (pI 4.81). A comparison of the deduced amino acid sequence of MT-I and MT-II indicates 25% identity.

**Figure 1:**
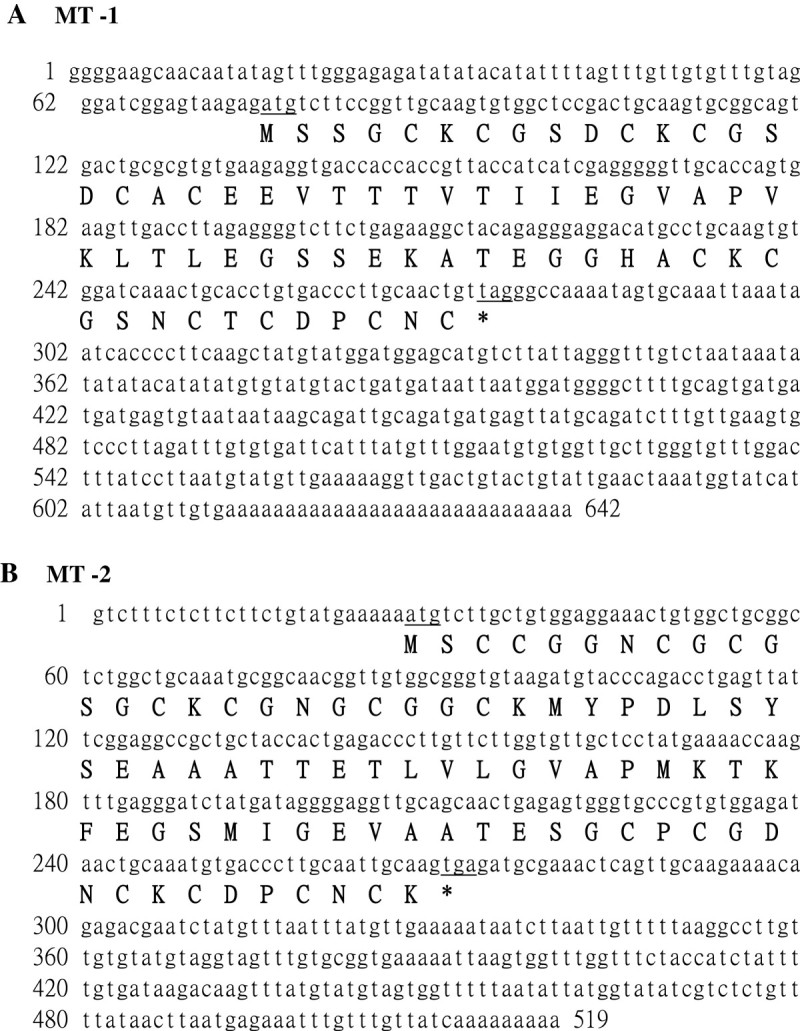
**DNA and amino acid sequences of two putative MTI and MT-II protein genes (MT-1, GenBank Accession Number AF116845 and MT-II, GenBank Accession Number FJ418632) isolated from sweet potato tuberous roots.** ATG (underlined) represents the start codon. TGA and TAA (underlined) are the stop codons for MT-1 and MT-II, respectively.

In plants, the members of MT family have been divided into four types according to the location and distribution of Cys residues. MT types 1–3 contain two Cys-rich clusters respectively at their N- and C-terminal regions, separated by a central Cys-free spacer of 30–40 residues. The type 4 which is known as Ec-type, has three Cys-rich clusters each separated by 10–15 residues (Nezhad, et al., [2013]). In this manuscript, amino acid sequences of MT-I and MT-II were compared at their N-terminal (domain 1) and C-terminal (domain 2) regions. The result showed that the deduced amino acid sequence of MT-I have a high degree of similarity with type 1 MT-like proteins from other plants, including a central hydrophobic domain flanked by conserved cysteine-rich motifs (conserved domain 1 region: CxCxxxCxCxxCxC and conserved domain 2 region: CxCxxxCxCxxCxC). In addition, deduced amino acid sequence of MT-II also exhibits a high degree of similarity with type 2 plant MT-like sequences, with the typical cysteine-rich domains at the N-terminal (CCxxxCxCxxxxCxCxxxCxxC) and C-terminal region (CxCxxxCxCxxCxC), respectively (Branislav, et al., [2013]). The data of gene structure analysis also agreed with the data from the comparison of amino acid sequences (Figure 1).

### Copy numbers of MT-I and MT-II sequences in sweet potato

**Figure 2:**
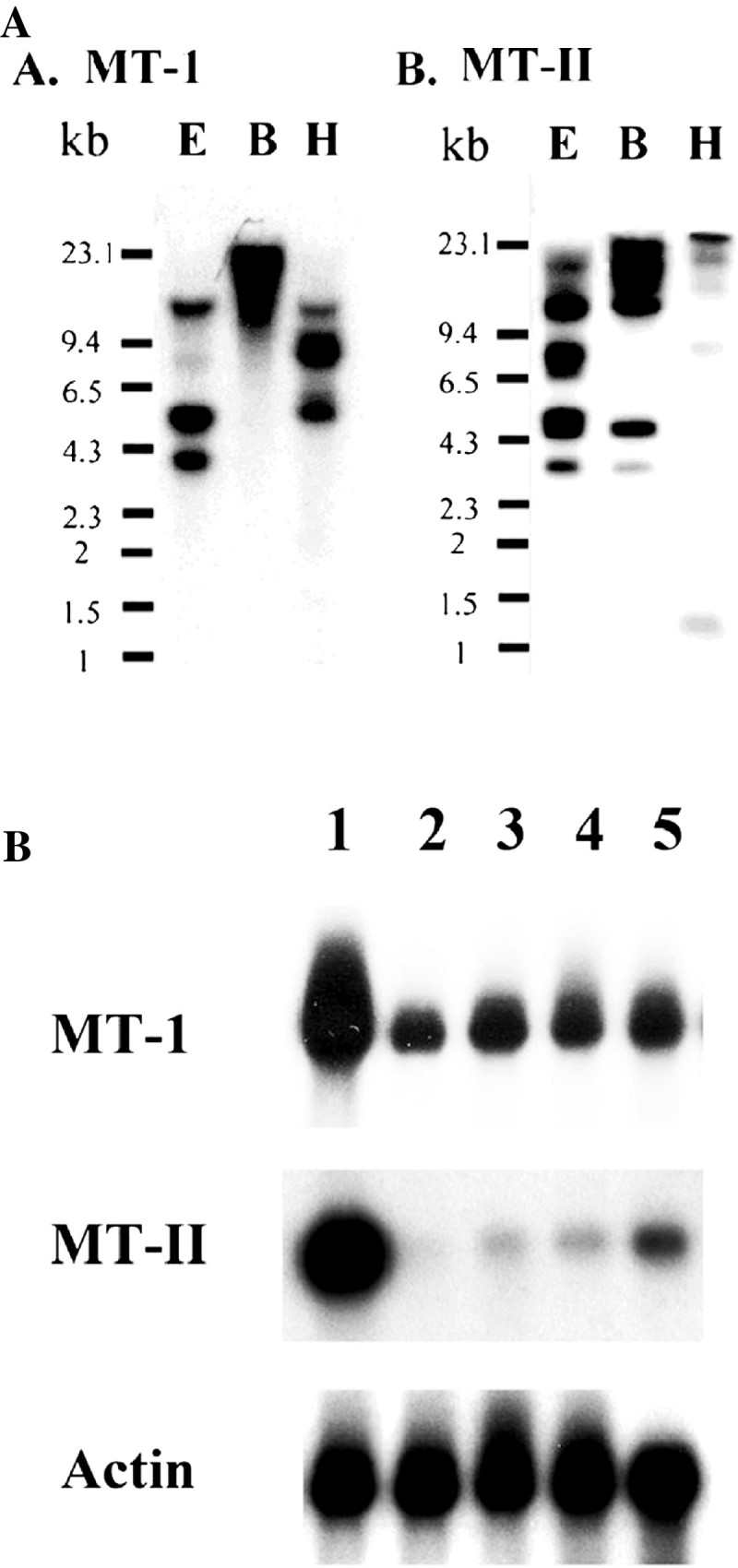
**Northern and Southern blot detections of MT-1 and MT-II genes. A.** Samples (10 μg) of genomic DNA from sweet potato Tainong 57 leaves were digested with *Eco RI* (E), *Bam HI* (B), and *Hind III* (H). The DNA fragments were separated in 0.8% agarose gels, transferred to a Hybond- N-nylon membrane, and hybridized with PCR-labeled cDNA probes. Molecular size markers were λ DNA/*Hind III* fragments. **B.** Northern blot analysis. Samples (10 μg each) of total RNA were isolated from different tissues of sweet potato and actin (AY905538) was utilized as an internal control of mRNA from sweet potato. Blots were hybridized to α-^32^P-labeled 3′ specific cDNA probes. Lane 1: storage roots, lane 2: sprout, lane 3: veins, lane 4: sprouted roots, and lane 5: full expanded green leaves. Actin was used as a control. The experiments were done twice and a representative one was shown.

We performed Southern blot hybridization with *Eco RI* (E), *Bam HI* (B) and *Hind III* (H) digests of sweet potato Tainong 57 DNA, using probe derived from 3′-noncoding sequence of the cDNAs to estimate the copy number of the gene. Tainong 57, an elite sweet potato cultivar derived from a cross between Tainong 27 and Nancy Hall, has a hexaploid number of chromosome (2n = 6x = 90). The results suggest that *MT-I* and *MT-II* belong to a small multigene family in sweet potato (Figure 2A).

### MT-I and MT-II mRNA levels were developmentally regulated

The presence and amounts of different sweet potato MT-I and MT-II mRNAs were examined in various organs and tissues by northern blot analysis. *MT-I* and *MT-II* were obtained from sweet potato storage roots. Figure 2B shows that *MT-I* and *MT-II* probe hybridized to mRNA species of approximately 1.0 kb. *MT-I* mRNA levels were the highest in the storage roots, followed by that in sprouted roots, fully expanded green leaves and vein; while it was the lowest in sprout. *MT-II* mRNA levels were the highest in the storage roots, followed by that in fully expanded green leaves; while it was the lowest in sprouted roots and vein.

### Expression of MT-I and MT-II in *E. coli*

**Figure 3:**
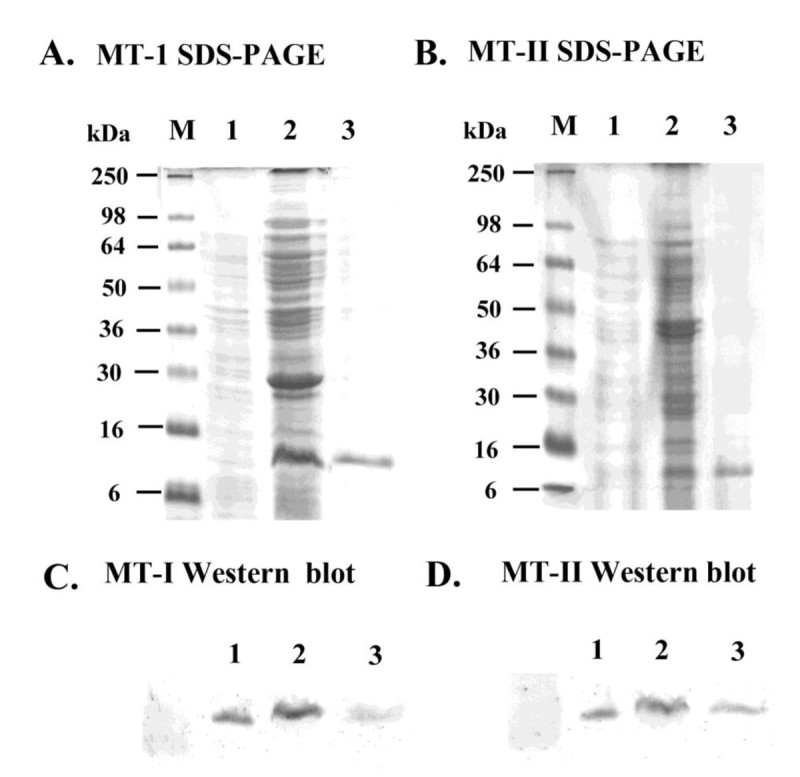
**Purified recombinant sweet potato MT-I and MT-II proteins. A.** MT-I proteins and **B.** MT-II proteins 15% SDS-PAGE analysis. Crude extracts (5 μg protein) from *E. coli* (M15) transformed with pQE30 (lane 1) or with pQE31- MT-I (lane 2) or pQE31- MT-II were analyzed by 15% (*w/v*) SDS-PAGE, and then the gels were stained with Coomassie brilliant blue G-250. Molecular masses of standard proteins were indicated at the left of the figure. His-tagged MT-I protein or MT-II was purified by Ni^2+^-chelated affinity chromatography (lane 3). **C.** MT-I proteins and **D.** MT-II proteins analyzed by Western blot. The gels were transferred onto PVDF membranes that were probed with a 1:1000 (*v/v*) dilution of mouse antibodies raised against MT using goat-anti-mouse alkaline phosphatase as the secondary antibody. The experiments were done twice and a representative one is shown. “M” indicated the see Blue™ pre-stained markers for SDS-PAGE. Each data show the mean ± SD of one experiment performed in triplicate.

SDS-PAGE analysis of MT-I and MT-II crude extracts from the transformed *E. coli* (M15) showed high amounts of a polypeptide with the expected molecular mass (ca. 6.5 and 8 kDa) (Figure 3A and 3B). Each polypeptide was found as a soluble protein in the supernatant (Figure 3A and 3B, lane 2), and was absent in protein extracts obtained from *E. coli* transformed with pQE-31 vector (Figure 3A and 3B, lane 1). The expressed protein was highly purified from crude extracts as His-tagged pQE-MT-1 and pQE-MT-II (Figure 3A and 3B, lane 3), respectively. The polypeptides of MT-I and MT-II were analyzed by western blot assay. As shown in Figure 3C and 3D, MT-I and MT-II proteins expressed in the transformed *E. coli* (M15).

### Effect of pH (6.0 and 7.0) on dehydroascorbate reductase activity of MT-I and MT-II proteins

**Figure 4:**
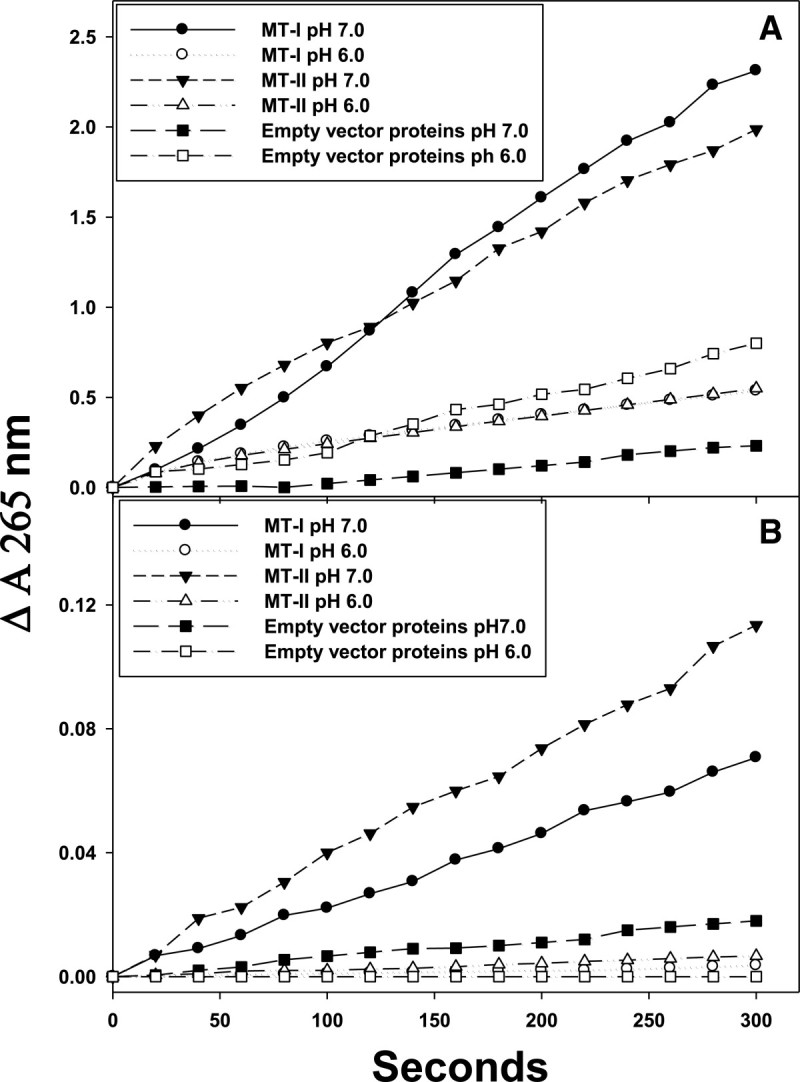
**Effect of pH (6.0 and 7.0) on dehydroascorbate reductase activity.** Purified recombinant protein of MT-I and MT-II were with **(A)** or without **(B)** 4 mM glutathione in the reaction mixtures. The reaction was carried out at 30°C by adding 100 μL MT-I and MT-II solution (100 μg protein, 100 mM phosphate buffer, pH 7.0 and 6.0) to 0.9 mL DHA solution with or without 4 mM glutathione. The increase of absorbance at 265 nm was recorded for 5 min. The experiments were done twice and a representative one is shown.

**Figure 5:**
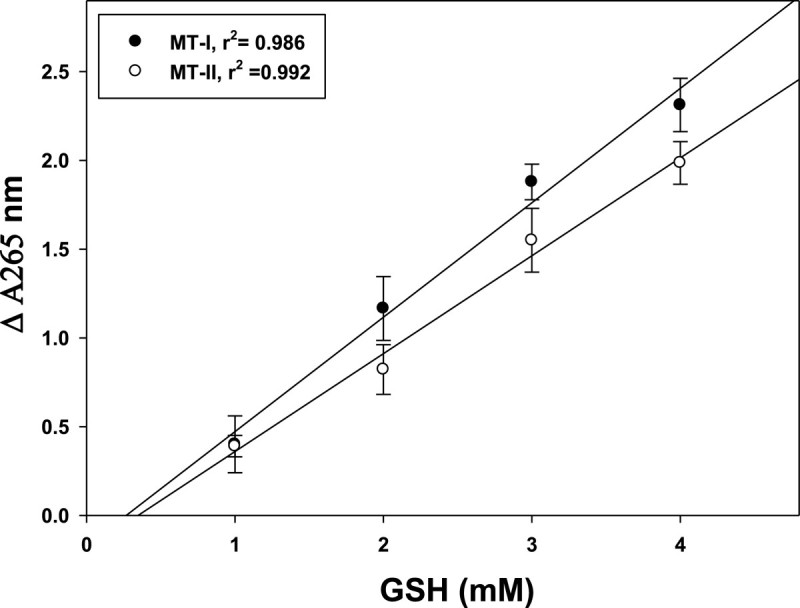
**Dependence of dehydroascorbate reductase activity of MT-I and MT-II on GSH concentration.** The reaction was carried out at 30°C by adding 100 μL MT-I and MT-II solution (100 μg protein, 100 mM phosphate buffer, pH 7.0) to 0.9 mL DHA solution with different concentrations of glutathione. The increase of absorbance at 265 nm was recorded for 5 min. Each data show the mean ± SD of one experiment performed in triplicate.

The purified MT-I and MT-II were used to examine DHA reductase activity. Figure 4 shows AsA regeneration (ΔΑ 265 nm) from DHA at both pH 6.0 and 7.0 with (A) or without (B) GSH. Figure 4A shows that MT-I and MT-II exhibited DHA reductase activity and could reduce DHA back to AsA. The specific activities of DHA reductase for MT-I and MT-II in the presence of GSH were 3.45 and 5.52 nM AsA produced/min/mg protein at pH 7.0, respectively. However, in the absence of GSH, very low DHA reductase activities of MT-I and MT-II were found (Figure 4B): only 0.01 and 0.02 nM AsA produced/min/mg protein at pH 7.0, respectively. In addition, the specific activities of DHA reductase for MT-I and MT-II in the presence of GSH were 1.86 and 1.28 nM AsA produced/min/mg protein at pH 6.0, respectively. However, in the absence of GSH, very low DHA reductase activities of MT-I and MT-II were found (Figure 4B): only 0.006 and 0.018 nM AsA produced/min/mg protein at pH 6.0, respectively. MT-I and MT-II act as a GSH-dependent DHA reductase (Figure 5), and the rate of reduction was closely proportional to the concentration of GSH.

DHA is generated from the disproportionation of the MDA radical produced following the oxidation of ASA. DHA reductase catalyses the reduction of DHA to ASA using GSH as the reductant (Wu, et al., [2009]). If DHA is not recycled to ASA, it undergoes irreversible hydrolysis to 2, 3-diketogulonic acid. Expression of DHA reductase in plant, responsible for regenerating AsA from an oxidized state, regulates the cellular AsA redox state, which in turn affects cell responsiveness and tolerance to environmental reactive oxygen species (ROS). Because of its role in AsA recycling, we examined whether DHA reductase is important for plant growth (Wang, et al., [2010]). In its reaction with ROS, ASA is oxidized to the short-lived radical, MDA, which can rapidly disproportionate non-enzymatically to produce DHA and ASA. Alternatively, MDA can reduce DHA to ASA using NADPH as the reductant. Therefore, plants have evolved several mechanisms by which the oxidized forms of ASA can be recycled (Kerchev, et al., [2012]).

The most critical advance in MTs research is the demonstration of the redox regulation of Zn-S interaction and the coupling of zinc and redox metabolism (Oteiza, [2012]). The cluster structure of Zn-MT provides a chemical basis by which the cysteine ligand can induce oxidoreductive properties. The hypothesis that MT functions as an antioxidant against ROS and reactive nitrogen species has received extensive experimental support from many of the *in vitro* studies. Studies using a cell-free system have demonstrated the ability of MT as a free radical scavenger. MT has been shown to scavenge hydroxyl radical *in vitro*, because of its cysteinyl thiolate groups (Miura, et al., [1997]). In ad dition, there are possible reasons to explain the apparent low DHAR activity of MT-I and MT-II. Zinc (II) is an important regulator of GSH synthesis. The importance of zinc in the metabolism of GSH underscores the finding that, as zinc deficiency is accompanied by oxidant increase, many studies reveal a deficiency of GSH under such conditions (Hernandez, et al., [2012]). Therefore, MT-I and MT-II may be less reduced by GSH resulting in low DHAR activity comparing to other DHAR.

### Effect of pH (6.0 and 7.0) on monodehydroascorbate reductase activity of MT-I and MT-II proteins

**Figure 6:**
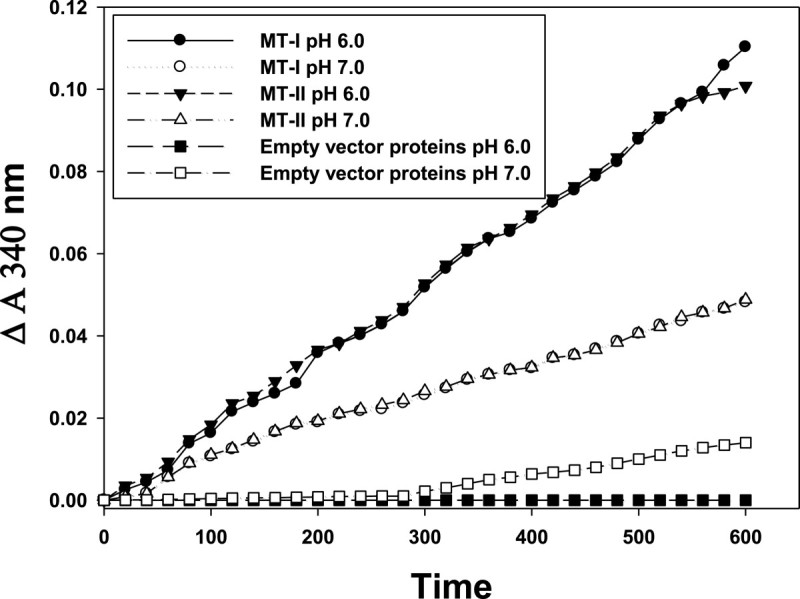
**Effect of pH (6.0 and 7.0) on monodehydroascorbate reductase activity of MT-I and MT-II.** The reaction mixtures contained 50 mM phosphate buffer (pH 6.0 and 7.0), 0.33 mM NADH, 3 mM AsA, AsA oxidase (0.9 U), and 200 μL MT-I and MT-II solution (200 μg protein) in a final volume of 1 mL. MT-I and MT-II solution was replaced with distilled water for controls. The experiments were done twice and a representative one is shown.

MDA was reduced to AsA in coupling with NADH oxidation (Δ A340 nm) at pH 6.0, and 7.0 when MT-I and MT-II proteins was used as MDA reductase. The MT-I and MT-II proteins exhibited MDA reductase activity at both pH 6.0 and 7.0 (Figure 6), with higher activity at pH 6.0 than pH 7.0. Therefore, the specific MDAR activity of MT-I and MT-II proteins was 0.18 and 0.17 unit/mg protein in pH 6.0, respectively.

### Protein and diaphorase activity stainings in 15% SDS–PAGE gels for detection of monodehydroascorbate reductase activity of MT-I and MT-II proteins

**Figure 7:**
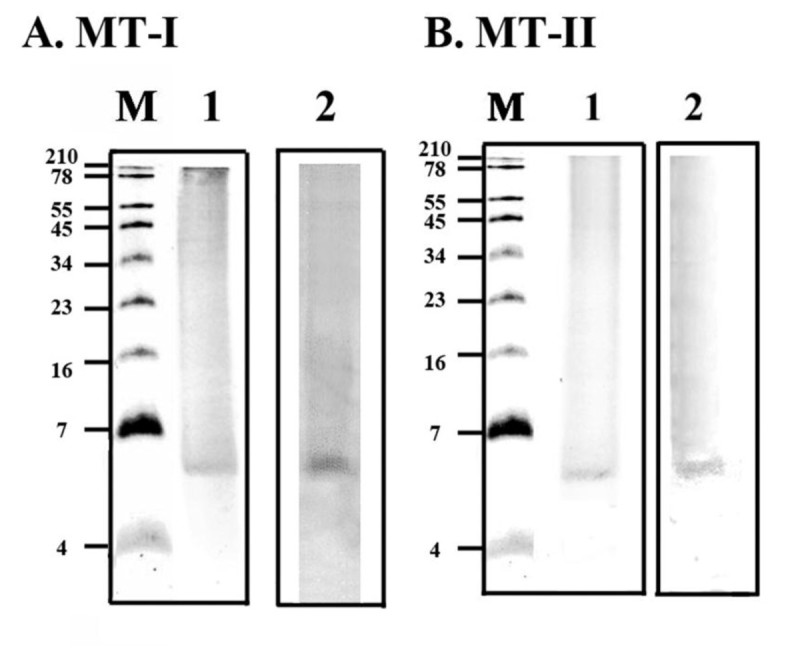
**Protein and diaphorase activity stainings in 15% SDS–PAGE gels for detection of monodehydroascorbate reductase activity of MT-I (A) and MT-II (B).** The experiments were done twice and a representative one is shown. ‘M’ represents the molecular weight marker and 10 μg proteins were loaded in each well.

MDA reductase activity staining of MT-I and MT-II was done for diaphorase activity (Kaplan and Beutler, [1967]) on SDS-PAGE gels (Figure 7). Comparing Figure 7 (A, lane 1 and B, lane 1) (protein staining) with Figure 7(A, lane 2 and B, lane 2) of MT-I and MT-II one can see that the diaphorase activity staining for MDA reductase activity came from 6 or 8 kD MT-I or MT-II. MDA reductase and DHA reductase were shown to contain free thiol groups in their catalytic sites (Trümper, et al., [1994]). When AsA is the sole hydrogen donor, the AsA peroxidase, guaiacol peroxidase, and AsA oxidase can produce MDA (Hou, et al., [1999]). Nonenzymatic oxidations of AsA also produce MDA when cells were under oxidative stress (Hossain, et al., [1984]). DHA reductase that catalyses the reduction of DHA by GSH have been purified from rice, spinach, and potato (Dipierro and Borranccino, [1991]). Several other proteins such as glutaredoxins (thiol transferases), protein disulphide isomerases, defensin, thioredoxin, and even a Kunitz-type trypsin inhibitor have been shown to have DHAR activity (Huang et al., [2008]; Huang et al., [2008]b). Plant Kunitz-type trypsin inhibitor has slight DHAR activity in its reduced form (Trümper et al. [1994]). Thioltransferase (glutaredoxin) and protein disulfide isomerase from animal cells also have DHAR activity (Wells et al. [1990]). Nevertheless, the amino acid sequence of the MT is quite distinct from these other DHAR enzymes.

## Conclusions

DHA reductase and MDA reductase activities of plant MT have been the subject of intensive study. However, little information is known about whether MTs also have DHA or MDA activity *in vitro*. Thus, that MT-I and MT-II cloned from storage roots of sweet potato appear to possess both DHA reductase and MDA reductase activities is an important finding. It becomes that MT-I and MT-II are suitable candidates to transform plants to improve resistance against various oxidative stresses. It also seems beneficial for people who consume sweet potato roots.

## Authors’ original submitted files for images

Below are the links to the authors’ original submitted files for images. Authors’ original file for figure 1 Authors’ original file for figure 2 Authors’ original file for figure 3 Authors’ original file for figure 4 Authors’ original file for figure 5 Authors’ original file for figure 6 Authors’ original file for figure 7
