# Supplementary material for: Dehydroascorbate reductase and monodehydroascorbate reductase activities of two metallothionein-like proteins from sweet potato (Ipomoea batatas [L.] Lam. ‘Tainong 57’) storage roots
Source: Bot Stud. 2013 Aug 21;54:7. doi: 10.1186/1999-3110-54-7 (PMC5430376; doi:10.1186/1999-3110-54-7)
Supplement: Supplementary file 1 — Authors’ original file for figure 1 [file 40529_2013_9_MOESM1_ESM.pdf]

A MT -1

```
  1 ggggaagcaacaatatagtttgggagagatatatacatattttagtttgttgtgtttgtag
 62  ggatcggagtaagagatgtcttccggttgcaagtgtggctccgactgcaagtgcggcagt
                     M  S  S  G  C  K  C  G  S  D  C  K  C  G  S
122 gactgcgcgtgtgaagaggtgaccaccaccgttaccatcatcgaggggttgcaccagtg
    D  C  A  C  E  E  V  T  T  T  V  T  I  I  E  G  V  A  P  V
182 aagttgaccttagaggggtcttctgagaaggctacagagggaggacatgcctgcaagtgt
    K  L  T  L  E  G  S  S  E  K  A  T  E  G  G  H  A  C  K  C
242 ggatcaaactgcacctgtgacccttgcaactgttagggccaaaatagtgcaaattaaata
    G  S  N  C  T  C  D  P  C  N  C  *
302 atcaccccttcaagctatgtatggatggagcatgtcttattagggtttgtctaataaata
362 tatatacatatatgtgtatgtactgatgataattaatggatggggcttttgcagtgatga
422 tgatgagtgtaataataagcagattgcagatgatgagttatgcagatctttgttgaagtg
482 tcccttagatttgtgtgattcatttatgtttggaatgtgtggttgcttgggtgtttggac
542 tttatccttaatgtatgttgaaaaaggttgactgtactgtattgaactaaatggtatcat
602 attaatgttgtgaaaaaaaaaaaaaaaaaaaaaaaaaaaaa 642
```

B MT -2

```
  1  gtctttctcttcttctgtatgaaaaaatgtcttgctgtggaggaaactgtggctgcggc
                               M  S  C  C  G  G  N  C  G  C  G
 60 tctggctgcaaatgcggcaacggttgtggcgggtgtaagatgtacccagacctgagttat
    S  G  C  K  C  G  N  G  C  G  G  C  K  M  Y  P  D  L  S  Y
120 tcggaggccgctgctaccactgagacccttgttcttggtgttgctcctatgaaaaccaag
    S  E  A  A  A  T  T  E  T  L  V  L  G  V  A  P  M  K  T  K
180 tttgagggatctatgataggggaggttgcagcaactgagagtgggtgcccgtgtggagat
    F  E  G  S  M  I  G  E  V  A  A  T  E  S  G  C  P  C  G  D
240 aactgcaaatgtgacccttgcaattgcaagtgagatgcgaaactcagttgcaagaaaaca
    N  C  K  C  D  P  C  N  C  K  *
300 gagacgaatctatgtttaatttatgttgaaaaataatcttaattgtttttaaggccttgt
360 tgtgtatgtaggtagtttgtgcggtgaaaaattaagtggtttggtttctaccatctattt
420 tgtgataagacaagtttatgtatgtagtggtttttaatattatggtatatcgtctctgtt
480 ttataacttaatgagaaatttgtttgttatcaaaaaaaaa 519
```
