# Supplementary figures and images for: Dehydroascorbate reductase and monodehydroascorbate reductase activities of two metallothionein-like proteins from sweet potato (Ipomoea batatas [L.] Lam. ‘Tainong 57’) storage roots
Source: Bot Stud. 2013 Aug 21;54:7. doi: 10.1186/1999-3110-54-7 (PMC5430376; doi:10.1186/1999-3110-54-7)

**A****A. MT-1****B. MT-II**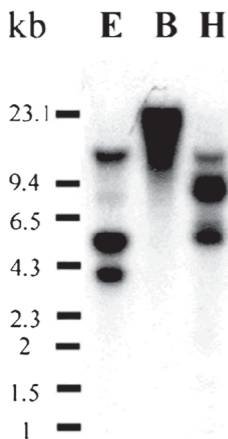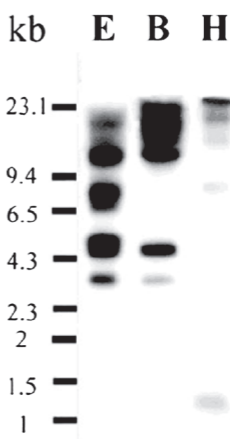**B****1      2      3      4      5****MT-1****MT-II****Actin**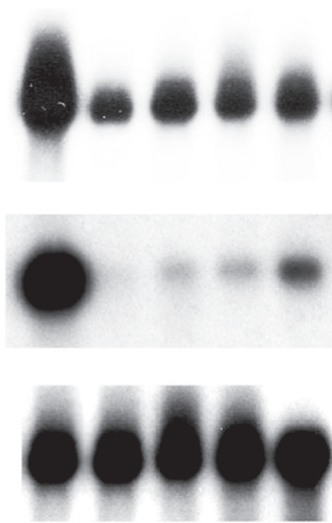

Supplement: Supplementary file 2 — Authors’ original file for figure 2 [file 40529_2013_9_MOESM2_ESM.pdf]

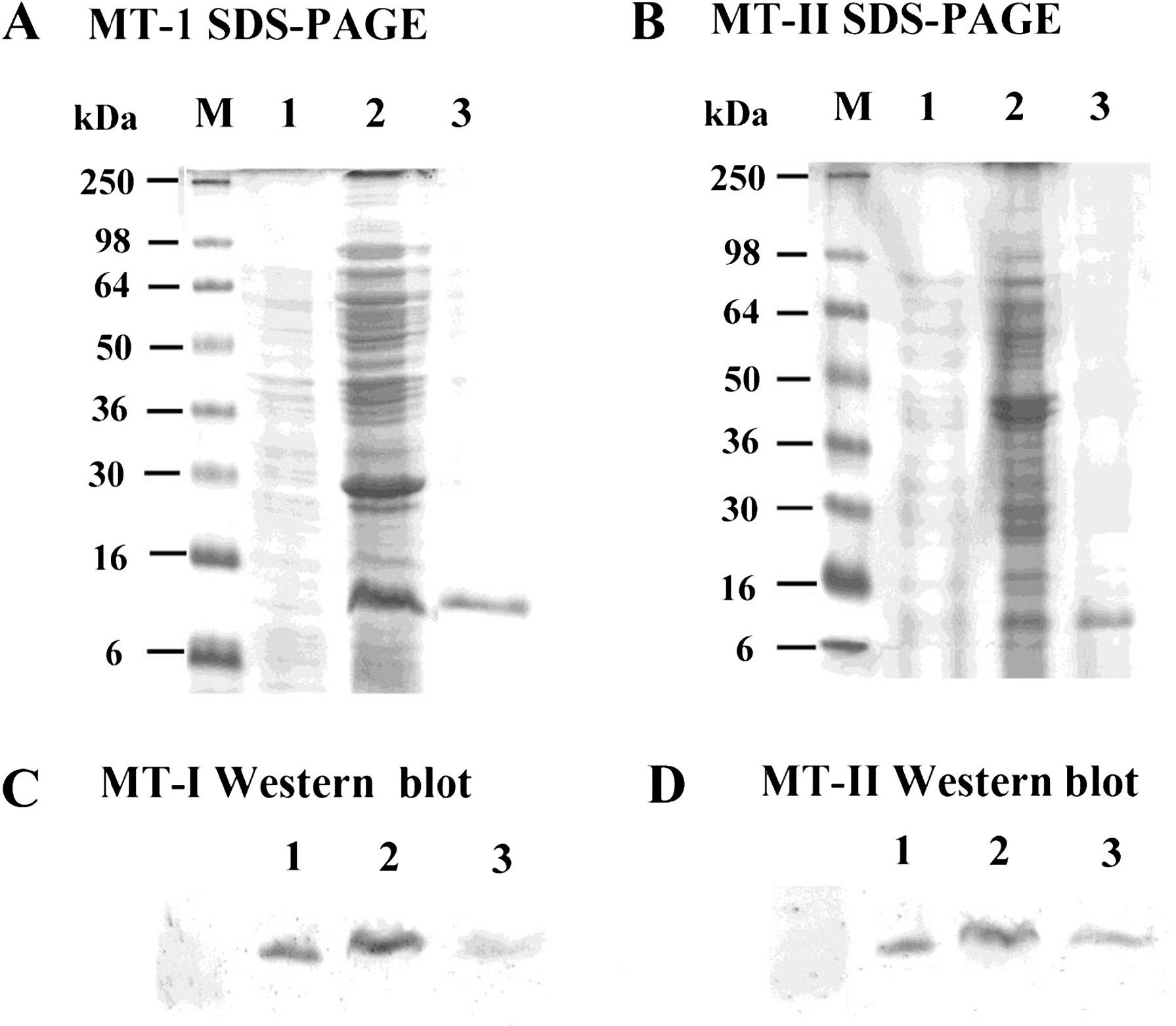

Supplement: Supplementary file 3 — Authors’ original file for figure 3 [file 40529_2013_9_MOESM3_ESM.tiff]

$\Delta A_{265\text{ nm}}$

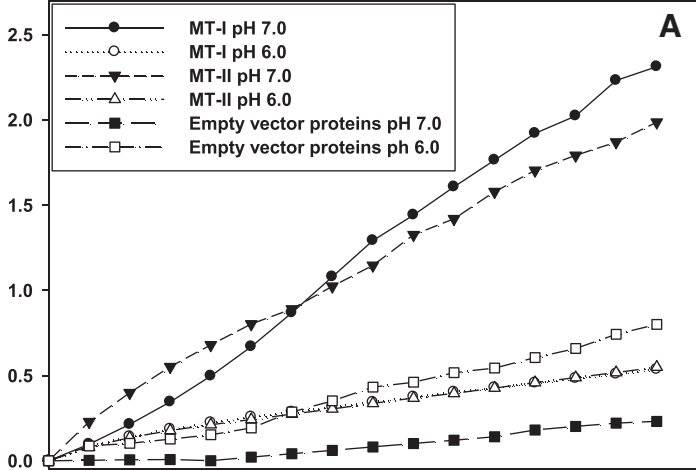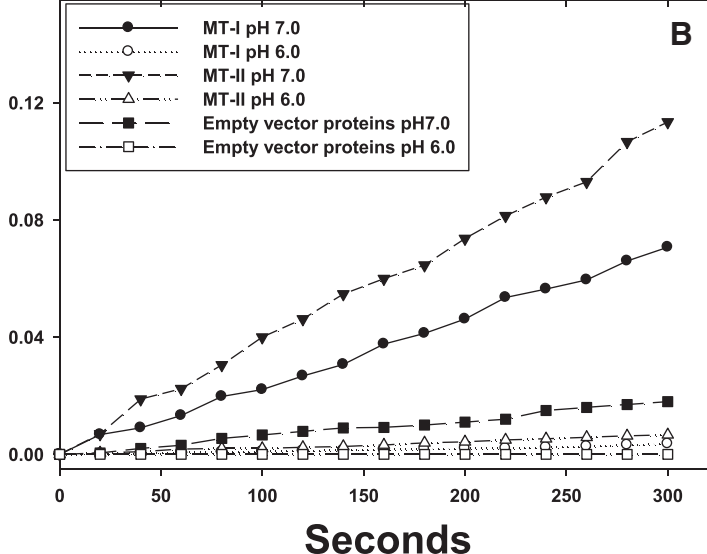

Supplement: Supplementary file 4 — Authors’ original file for figure 4 [file 40529_2013_9_MOESM4_ESM.pdf]

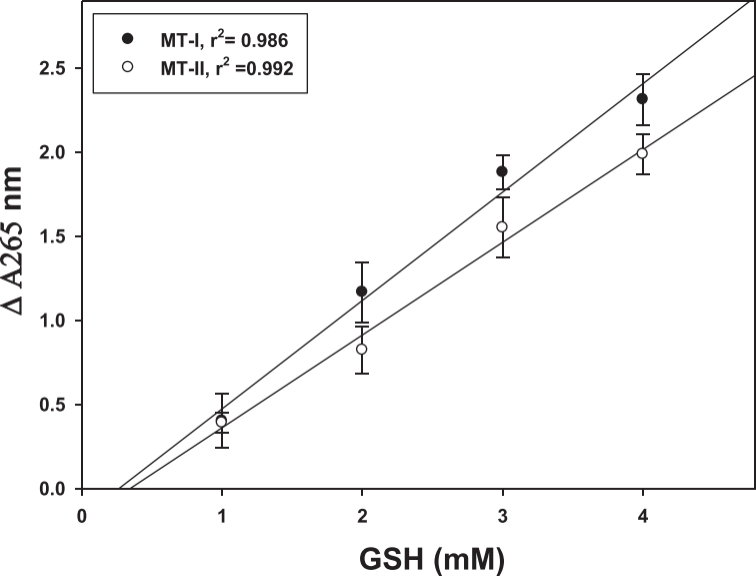

Supplement: Supplementary file 5 — Authors’ original file for figure 5 [file 40529_2013_9_MOESM5_ESM.pdf]

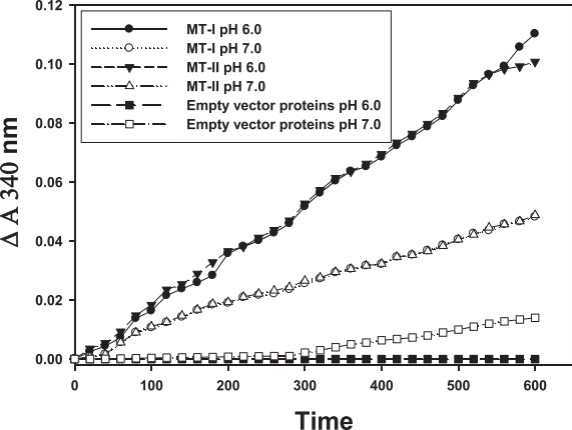

Supplement: Supplementary file 6 — Authors’ original file for figure 6 [file 40529_2013_9_MOESM6_ESM.pdf]

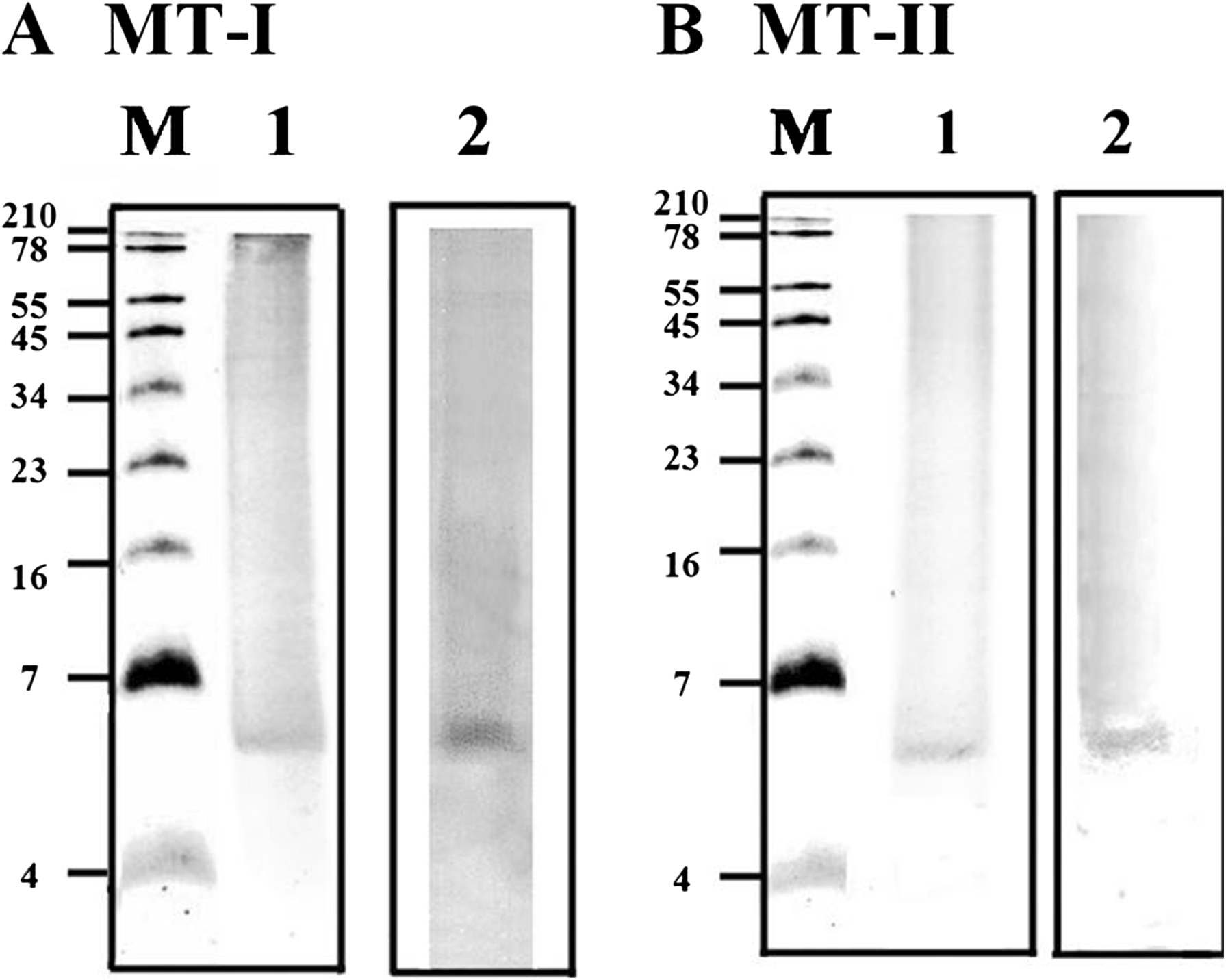

Supplement: Supplementary file 7 — Authors’ original file for figure 7 [file 40529_2013_9_MOESM7_ESM.tiff]
